# Genetic insights into immune mechanisms of Alzheimer’s and Parkinson’s disease

**DOI:** 10.3389/fimmu.2023.1168539

**Published:** 2023-06-08

**Authors:** Alexi Nott, Inge R. Holtman

**Affiliations:** ^1^ Department of Brain Sciences, Imperial College London, London, United Kingdom; ^2^ UK Dementia Research Institute, Imperial College London, London, United Kingdom; ^3^ Department of Biomedical Sciences of Cells and Systems, Section Molecular Neurobiology, University of Groningen, University Medical Center Groningen, Groningen, Netherlands

**Keywords:** microglia, immune, Alzheimer’s disease, Parkinson’s disease, GWAS - genome-wide association study, epigenetics, genetics

## Abstract

Microglia, the macrophages of the brain, are vital for brain homeostasis and have been implicated in a broad range of brain disorders. Neuroinflammation has gained traction as a possible therapeutic target for neurodegeneration, however, the precise function of microglia in specific neurodegenerative disorders is an ongoing area of research. Genetic studies offer valuable insights into understanding causality, rather than merely observing a correlation. Genome-wide association studies (GWAS) have identified many genetic loci that are linked to susceptibility to neurodegenerative disorders. (Post)-GWAS studies have determined that microglia likely play an important role in the development of Alzheimer’s disease (AD) and Parkinson’s disease (PD). The process of understanding how individual GWAS risk loci affect microglia function and mediate susceptibility is complex. A rapidly growing number of publications with genomic datasets and computational tools have formulated new hypotheses that guide the biological interpretation of AD and PD genetic risk. In this review, we discuss the key concepts and challenges in the post-GWAS interpretation of AD and PD GWAS risk alleles. Post-GWAS challenges include the identification of target cell (sub)type(s), causal variants, and target genes. Crucially, the prediction of GWAS-identified disease-risk cell types, variants and genes require validation and functional testing to understand the biological consequences within the pathology of the disorders. Many AD and PD risk genes are highly pleiotropic and perform multiple important functions that might not be equally relevant for the mechanisms by which GWAS risk alleles exert their effect(s). Ultimately, many GWAS risk alleles exert their effect by changing microglia function, thereby altering the pathophysiology of these disorders, and hence, we believe that modelling this context is crucial for a deepened understanding of these disorders.

## 1 Introduction

Microglia, the resident macrophages of the central nervous system, are uniquely adapted to the brain microenvironment (1, 2) and play an essential role in maintaining the health of the brain in development, cognition and plasticity (3). As with other tissue-resident macrophages, microglia are critically dependent on the lineage-determining transcription factor PU.1 (coded by the *SPI1* gene) and the colony-stimulating factor 1 receptor (CSF1R) (4). The functions of microglia are essential for maintaining brain homeostasis, such as phagocytosis of pathogens and cell debris, and they are responsive to inflammatory stimuli (5, 6). Microglia continuously survey the brain and provide a first line of defence against pathogens to protect injured neurons (7, 8). Furthermore, microglia are crucial for sculpting neural circuits and synapse formation during brain development (8, 9). Studies have shown that microglia play a role in the phagocytosis of synapses, which helps to shape the structural and functional connectivity of neural circuits (8, 10). In addition, microglia respond to a wide range of disturbances in the local brain microenvironment through antigen detection using an extensive array of cell surface receptors (11). Recent findings have indicated that microglia are essential for the growth and integrity of myelin, the white matter tracts of the brain that are formed by oligodendrocytes (12, 13). These findings build on work demonstrating that microglia exhibit temporal and spatial heterogeneity across development and between brain regions, as well as sex-associated differences (14–19). In summary, microglia are important for maintaining a healthy central nervous system and are potential therapeutic targets for neurodegeneration and ageing.

**Figure 1:**
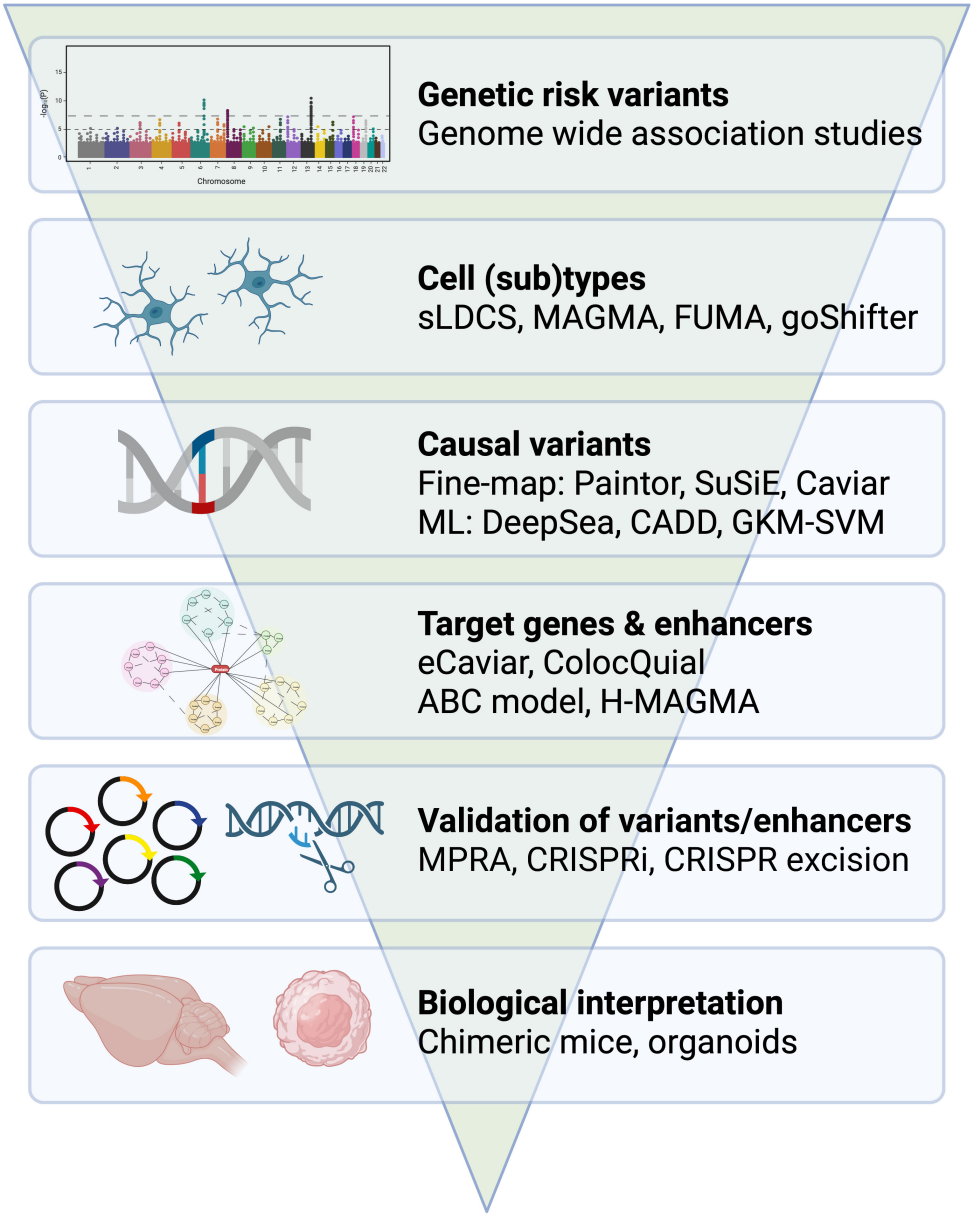
From GWAS to immune mechanisms. Overview of genetic approaches and tools that examine the role of microglia in dementia. Example tools have been provided for the identification of cell (sub)types, casual variants, target genes and enhancers. Example approaches have been provided for the validation of variants and enhancers and for the biological interpretation of risk genes.

Microglia have been increasingly recognized as key players in the development and progression of neurodegenerative and neuroinflammatory disorders, including AD (20–22), PD (23, 24), and multiple sclerosis (MS) (25, 26). Several studies suggest that microglia are activated and acquire an aberrant phenotype in the early stages of these disorders and contribute to the inflammatory and neurodegenerative processes that occur in the brain (27). Additionally, genetic studies have identified specific genetic loci that are associated with susceptibility to these disorders and implicate microglia in their pathogenesis (28). Understanding microglial biology and its role in neurodegeneration is an ongoing area of research, which has sparked an interest in immune modulation as a therapeutic strategy for treating dementia (29, 30). In this review, we will examine the genetic mechanisms that involve microglia in the development of AD and PD and provide an overview of the key concepts and techniques used in genome-wide association studies (GWASs) and post-GWAS analysis (**Figure 1**). While this review is focused on the interpretation of noncoding GWAS variants on microglia function, the contribution of rare coding variants has been previously discussed for AD and PD (31, 32).

GWAS is a powerful tool for identifying common genetic variants, also known as single-nucleotide polymorphisms (SNPs), that are associated with a trait. These traits can range from quantitative traits such as intelligence to qualitative traits such as type-1 diabetes (33). GWASs investigate complex traits in which many common variants, usually with small effect sizes, play a role in disease vulnerability. The power to detect genome-wide significant SNPs that are associated with a trait is largely dependent on sample size, and large-scale sample sizes are increasingly being obtained through meta-analyses of multiple cohorts (34–36). While GWASs are useful in identifying common variants associated with a phenotype, they do not provide insight into the causal cell types. Additionally, most GWAS-significant variants are in non-coding parts of the genome and in linkage disequilibrium (co-inherited) with the causal variant, leading to a number of challenges in GWAS interpretation. These include identification of the target cell type(s) and cell state(s) of GWAS risk loci, determining which variants are casual and how they exert their effect and identifying the genes and regulatory elements that mediate the risk. Integrative (epi)genomic and genetic analysis can address these challenges and provide informed decisions on how to perform validation experiments that explore the molecular function of these variants (**Figure 1**).

## 2 Alzheimer’s disease

### 2.1 AD and the role of microglia in pathophysiology

AD is a progressive, neurodegenerative disorder of the brain that affects memory, thinking, and behaviour. It is the most common cause of dementia and is characterised by the abnormal accumulation of extracellular amyloid-β (Aβ) plaques and intracellular neurofibrillary tangles composed of hyperphosphorylated tau protein (37, 38). These pathological changes are thought to be a result of an interaction between genetic, environmental and lifestyle factors. In the earliest stages of the disease, inflammation and oxidative stress are involved in the pathology of AD and play a role in cognitive decline and neurodegeneration (39–41).

Genomic studies using bulk brain tissue have identified microglia dysregulation at the core of AD pathogenesis through examining multiple elements of transcriptional regulation, including gene expression (42, 43), histone modifications (44, 45) and the proteome (46, 47). Dysregulated gene regulatory regions were identified in a mouse model of neurodegeneration near immune-related genes and were predicted to bind the myeloid PU.1 transcription factor (44). These dysregulated gene regulatory regions proximal to immune genes were enriched for AD GWAS risk variants at orthologous regions in humans (44). In human AD, dysregulated gene regulatory regions were identified using cortical bulk tissue (48–50) and cell type enriched populations (45) and were located near familial AD genes (*APP*, *PSEN1* and *PSEN2*) and *MAPT* that codes for tau protein. Gene regulatory regions that were dysregulated in AD were associated with microglia, however, intriguingly, more pronounced changes were found in oligodendrocytes (45, 51).

Single nuclei transcriptome studies have explored heterogeneity across subpopulations of cells in brain disorders including AD. Partially overlapping disease-associated-microglia (DAM) gene expression signatures were identified across AD, ageing and other brain disorders in human and mouse models (14, 52–60). Additional microglia subpopulations have been observed in disease conditions, including type I interferon (IFN1), major histocompatibility complex class II (MHCII), proliferative, and tau-associated subsets (53, 61). However, the functional consequences of these microglia subsets are still largely unknown. Spatially, alterations in cell state have been observed in microglia near amyloid plaques and microglia in the white matter prior to amyloid deposition (62, 63). Additional myeloid cell types that may also play a role in AD pathogenesis include infiltrating monocytes (64), choroid plexus macrophages (65), perivascular macrophages (66, 67) and border-associated macrophages (68). The immune contribution of common genetic variants for brain disorders has largely focused on microglia because they are the predominant brain resident macrophage. However, it should be considered that genetic risk is likely to be shared across multiple myeloid cell types, and conversely, the impact of genetic risk may change following transitions in cell state.

The protective and neurodegenerative role of microglia is complex and depends on the disease type, stage, specific pathogenic features, and transcriptional responses. For example, microglia that surround amyloid beta plaques were described as proliferative and activated and were suggested to restrict amyloid beta propagation and toxicity, thereby exerting a protective role (69, 70). However, microglia that were described as activated in AD can become harmful by secreting inflammatory mediators and engulfing healthy synapses (69).

### 2.2 AD Genetics

Rare variants that cause autosomal dominant forms of AD were found in *PSEN1*, *PSEN2*, and *APP* genes in familial cases of AD (71–73). These genes contributed to the amyloid hypothesis, which proposed that amyloid-beta aggregation is a primary cause of AD progression (74, 75). Subsequent studies identified rare coding variants associated with an increased risk for AD in genes that are expressed in microglia, including *TREM2*, *PLCG2* and *ABI3* (76–78). The selective expression of AD risk genes in microglia suggested a possible causative role for brain immunity in AD susceptibility.

Further insights into the genetics of AD have been provided by GWASs. A 2013 AD GWAS that included a meta-analysis of 74,000 individuals identified 11 AD risk loci (79). This study identified a shared genetic basis between sporadic and familial AD (79). Subsequent AD GWASs have been conducted, including a recent meta-analysis of 788,989 individuals that identified 75 risk loci (34). Later GWASs included many by-proxy cases (relatives of affected individuals), which may have affected the reliability of the results (80). Notably, the latest AD GWAS identified risk loci that may share causal variants with frontotemporal dementia (34), which may be influenced by the inclusion of proxy AD cases without a clinical diagnosis. A definitive diagnosis of neurodegenerative disorders, including AD, can only be made through neuropathological examination. Neuropathological examinations showed that up to a third of clinically defined AD cases were incorrectly diagnosed, which could potentially skew the results of GWASs (81, 82). Twin studies suggest a heritability of 58-79% for late-onset AD (83) and 90% for early-onset AD (84). GWASs suggest a SNP heritability of 38-66% (85), which indicates that there is still missing heritability that cannot be explained by significant common genetic variants alone.

### 2.3 Post-AD GWAS: target cell types and cell states

GWAS-risk loci are often located in non-coding regions of the genome. Identifying the target cell types and states is frequently performed by studying cell type-genomic datasets, gene expression profiles, and maps of gene regulatory elements through profiles of open chromatin or histone modifications. In the early GWASs, genes were assigned to loci based on genomic distance, however, GWASs now often incorporate cell-type gene expression datasets and other genomic datasets to link individual loci to genes using functional mapping tools such as FUMA (86), or global enrichment tools such as MAGMA (87). Single-cell sequencing technologies have provided gene expression profiles for a wide range of human brain cell subtypes. Nonetheless, many genes are expressed in multiple cell types, and GWAS risk alleles can regulate the expression of more distant genes. Non-coding GWAS variants are often found in gene regulatory regions, such as promoters and enhancers (88). Enhancers are short regions of DNA that, when activated by transcription factors, can regulate gene expression, sometimes at a considerable distance. Enhancers are specific to certain cell types, and most are only active in a small subset of tissues and/or cell types (89). Putative enhancers can be identified by distinct post-translational modifications of histone proteins that package DNA into chromatin and are associated with increased chromatin accessibility. Stratified linkage disequilibrium score (sLDSC) regression analysis was developed to identify the target cell types/states of specific GWASs by performing a GWAS heritability enrichment analysis (90).

Early studies identified an enrichment of myeloid cells for AD genetic risk using macrophage gene expression profiles (90–92). Subsequently, it was found that AD GWAS risk genes had higher gene expression levels in human microglia compared to bulk brain, and many of these genes were downregulated in cultured microglia (93). Later studies found that AD heritability was enriched in regions that surround microglia-expressed genes (34, 35, 94–96). Of note, cell type enrichment based on gene expression profiles often extend the genomic regions around a gene by a defined distance, however, many non-coding regulatory elements may be located more distal to the gene and potentially missed. Correspondingly, studies using sLDSC regression analysis found an enrichment of AD heritability in gene regulatory elements of monocytes and myeloid cells (90, 92, 97). Similarly, human microglia showed clear enrichment of AD heritability in gene regulatory regions, which was more pronounced for enhancers than promoters (98–100). These findings indicate that microglia and possibly other cell types in the myeloid lineage are causally implicated in the pathogenesis of AD.

A major difficulty in understanding the genetic risk of AD is that related cell types share genomic features, making it difficult to identify the specific cell types and states that may be affected. For example, it can be challenging to distinguish whether the enrichment of myeloid cells and monocytes in AD is caused by shared epigenetic features with microglia, or if AD risk loci directly influence multiple myeloid cell types. In addition, global enrichment of GWAS genetic heritability for a particular cell type does not mean that genetic susceptibility at individual loci are not important in other cell types. Therefore, it is important to consider how each locus might influence relevant cell types and cell states.

### 2.4 Post-AD GWAS: causal variants and their mechanisms

A second major challenge for GWAS interpretation is identifying which variants are causal at each locus and understanding the mechanism by which their effect is mediated. This can be difficult to achieve due to the non-random association of neighbouring alleles, known as linkage disequilibrium (LD). Most genome-wide significant variants, often in the range of tens to hundreds of variants in a locus, are thought to be non-functional but are in high LD with one or a few causal variant(s). Additionally, the causal SNPs may not have been genotyped or imputed, and hence not included from the analysis, or the LD block might be driven by other genomic features such as indels, repetitive elements, or rare variants (101). Taken together, these levels of complexity make it difficult to identify true causal variants, which are a figurative needle in the haystack.

There are multiple strategies to identify putative causal variants, which often implement fine-mapping and machine learning-based prioritisation tools. Fine-mapping approaches have been developed to identify putative causal variant(s) for individual GWAS loci by integrating the LD structure from a reference genome as a Bayesian prior. The output of fine-mapping tools is referred to as a ‘credible set’ that with a particular percentage of confidence (often set to 95%) should contain the causal variant. More recent fine-mapping tools can integrate genomic features to identify putative causal variants with higher reliability (102). Of note, the user selects the genomic features and the decision to include a particular set is frequently based upon sLDSC regression enrichment scores.

Another set of tools used to identify putative causal variants are based on machine learning algorithms that are trained on the genomic sequences associated with various genomic features. These machine learning prioritisation tools, such as DeepSea (103) and CADD (104), often use cell-type specific genomic sequences, such as gene regulatory elements, to build models that predict whether a given DNA sequence belongs to a functional annotation, as a binary classification task. By using these models to calculate a prediction score for both the reference and the alternative sequence, a delta score can be calculated, which is indicative of the ability of the variant to perturb a regulatory element or transcription factor binding site. These machine learning models have been successfully applied in the context of variant prioritisation for AD (95, 99, 105).

**Figure 2:**
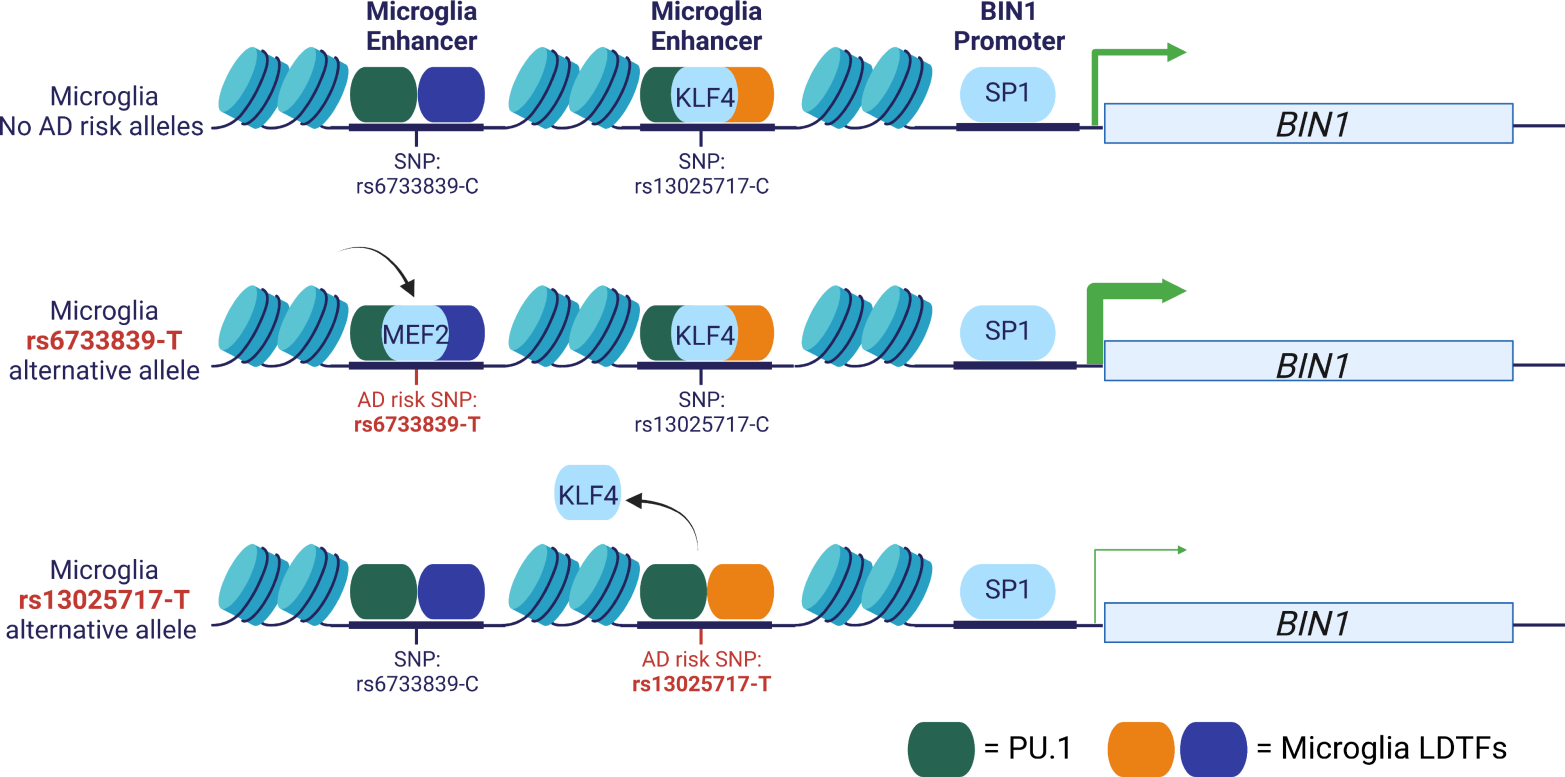
Example AD GWAS risk variants at the *BIN1* locus. Two AD GWAS risk variants, rs6733839 and rs13025717, are localized to chromatin-accessible regions at two microglia enhancers (98, 99). CRISPR excision of either enhancer reduces *BIN1* expression in iPSC-derived microglia-like cells (98, 106). The rs6733839-T risk variant is predicted to create a MEF2 binding motif and the rs13025717-T risk variant is predicted to alter a KLF4 binding motif and both variants are located adjacent to PU1 binding sites (97, 99). The rs6733839-T variant is an eQTL in human microglia and the rs6733839-T is an MPRA functional regulatory variant in HEK293 cells (106, 107). Both variants have been associated with differences in chromatin accessibility in human brain and rs6733839-T in iPSC-derived microglia-like cells (97, 107).

Schwartzentruber and colleagues performed fine-mapping on an AD GWAS using multiple tools and applied DeepSea to prioritise variants (95). They identified 21 SNPs with a >50% probability of being the causal variant and 79 additional variants with a probability of >10% to 50%. One of these variants is rs6733839, which is located upstream of the *BIN1* gene and colocalizes with a microglia enhancer (98) (**Figure 2**). The *BIN1* rs6733839 variant has been predicted to introduce a binding site for the transcription factor MEF2 (97, 107) and *BIN1* gene expression has been shown to be increased in AD brains (108). The MEF2 transcription factor is important for the formation of the microglia gene regulatory landscape (2). Additional evidence for the importance of this variant comes from an allelic imbalance in chromatin accessibility in induced pluripotent stem cell (iPSC)-derived microglia-like cells (107) and human brain (97). A second potential causal variant has been identified at the *BIN1* locus, rs13025717 (97, 99), which resides at a neighbouring microglia enhancer (98) (**Figure 2**). The rs13025717 variant has been predicted to alter a KLF4 binding site (99), a transcription factor implicated in myeloid differentiation (109). The *BIN1* rs13025717 variant also demonstrates allelic imbalance in the human brain (97). A functional role of the rs13025717 variant on gene expression was provided by a massive parallel report assay (MPRA) performed in Human embryonic kidney 293 cells (HEK293) cells (106). The rs6733839 -containing gene regulatory region was demonstrated to be a microglia-specific enhancer by CRISPR-mediate excision of the gene regulatory region, which ablated *BIN1* expression in iPSC-derived microglia-like cells and not neurons or astrocytes (98). Subsequently, CRIPSR-mediate excision of the neighbouring rs13025717-containing enhancer similarly reduced *BIN1* expression in iPSC-derived microglia-like cells (106). These findings suggest that rs6733839 and rs13025717 are likely causal variants that regulate *BIN1* expression (**Figure 2**). However, other putative AD causal variants do not show similar consistency across data types and analysis methods, andthe causal variants and mechanisms of action of most GWAS risk alleles remain a topic of debate.

Corces et al. trained a machine learning model, the gapped-kmer SVM (gkm-SVM), on single-cell chromatin accessibility data from major brain cell types (including microglia) (99). These trained models were used to identify putative causal variants in AD. One of the variants that was identified (rs10130373), was found to disrupt an *SPI1* motif in a microglia-specific open chromatin region at the *SLC24A4* locus (99). This disruption is noteworthy as the *SPI1* motif can be bound by the PU.1 transcription factor, which plays a critical role in microgliogenesis and the establishment of the microglial gene regulatory landscape (2, 4). Future single-cell epigenomic studies on brain-derived macrophages may reveal AD genetic risk variants that are localised to gene regulatory regions specific to myeloid subtypes and cell states.

In addition to analysing the impact of individual variants on specific transcription factor DNA binding motifs, other researchers have employed large-scale motif perturbation strategies to identify putative upstream transcription factors that may be dysregulated by multiple GWAS variants simultaneously (110, 111). For example, Kosoy et al. (100) used transcription factor footprinting to identify microglia-specific transcription factor DNA binding motifs and establish an AD-GWAS microglia gene regulatory network (100). In this network, the *SPI1* DNA binding motif was identified as the key upstream regulator. Other upstream DNA binding motifs were also identified, linked to an additional 15 transcription factors, some of which are associated with immune signatures (100).

Most current GWAS and post-GWAS studies presume that SNPs independently exert their effect on a given phenotype. A major reason for this simplification is that including SNP-SNP interactions results in a large multiple-testing burden and many potential different outcomes (112). However, several studies have shown that studying SNP-SNP interactions within the confines of relevant cell type-specific enhancers allows for the identification of sets of enhancer SNPs that cooperatively affect gene expression profiles (113–115), which highlights additional levels of complexity for some loci.

### 2.5 Post-AD GWAS: predict risk genes and regulatory elements

A major incentive for identifying causal GWAS variants is to determine the associated risk gene and the importance of the risk gene in the aetiology of the disease. There are two (non-mutually exclusive) converging lines of research that aim to link which genes and regulatory elements mediate GWAS risk loci: (1) chromatin looping and (2) quantitative trait locus (QTL) based approaches.

Enhancers have been proposed to mediate gene expression through physical contact with target gene promoters, often referred to as enhancer-promoter chromatin loops (116). Enhancers regulate target gene expression by binding transcription factors and chromatin regulators such as the mediator complex. Structural proteins bring enhancers in close proximity to target genes through DNA looping, which allows enhancers to regulate target genes from a distance. Chromatin interactions, including loops, can be detected genome-wide using a technique called Hi-C. However, the identification of enhancer-to-gene contacts can be challenging due to the high number of chromatin interactions that occur within a cell. To overcome this limitation, researchers use techniques such as HiChIP (117), PLAC-seq (118), and promoter-capture Hi-C (119) to capture chromatin interactions that are anchored to gene promoters and enrich for enhancer-to-gene contacts. Integrating chromatin interactions with enhancer annotations and gene expression data using the Activity-by-Contact (ABC) model can further improve the predictions of enhancer-to-gene contacts (120).

Nott et al. (98) used chromatin loops anchored to gene promoters of transcribed genes (H3K4me3-PLAC-seq) in microglia and other brain cell types and identified over 100,000 microglia chromatin interactions, including 20,000 enhancer-to-gene contacts (98). This study found that chromatin loops identified 50 genes that interacted with non-coding AD-risk variants, half of which were specific to microglia and not found in other neural cell types (98). These genes included fine-mapped high-confidence AD-risk variants linked to over 20 genes in microglia, such as *BIN1*, *PICALM*, *SPI1*, *TREM2*, *SORL1*, *USP6NL* and *ABCA7*. A subsequent study using single-cell chromatin looping data from the prefrontal cortex identified chromatin interactions linking AD-risk variants to *INPP5D* and *MS4A7* in microglia (121).

Corces et al. (99) used chromatin loops anchored to H3K27ac -enriched gene regulatory regions (H3K27ac-HiChIP) that were generated using bulk tissue from 6 brain regions and annotated to single-cell open chromatin regions (99). These cell-type-assigned chromatin loops were used to link disease-risk variants that overlap with microglia-specific open chromatin regions to target genes. The study identified *BIN1*, *MS4A6A* and *RIN3* as target genes for AD risk variants located within microglia-specific open chromatin regions.

Novikova et al. (97) integrated capture-based promoter chromatin loops with enhancers, and QTLs in monocytes and integrated this information with AD GWAS data to identify risk genes, under the assumption that chromatin architecture is relatively conserved across myeloid populations. This integrative approach identified candidate causal genes at 20 AD risk loci, including *AP4E1*, *AP4M1*, *APBB3*, *BIN1*, *MS4A4A*, *MS4A6A*, *PILRA*, *RABEP1*, *SPI1*, *TP53INP1* and *ZYX*. The study suggests that some loci might have multiple target genes co-regulated by enhancer-associated AD variants at the same locus.

Kosoy et al. (100) used Hi-C followed by deep sequencing of microglia from 5 individuals, which was integrated with distal open chromatin regions and gene expression data, to identify close to 25,000 high-confidence enhancer-to-gene contacts (100). The microglia enhancer-to-gene interactions were found to be enriched for AD risk variants. The study found that disease-risk regulatory regions were associated with the expression of a single gene for previously described genes such as *BIN1*, *PICALM*, *CD33*, *CASS4*, *ADAMTS4*, *INPP5D* and *APH1B*. Additionally, the study also found that previously unresolved loci such as *EPHA1-AS1*, *USP6NL*, *CCDC6*, *AC099524.1*, *ZNF652*, *MS4A4E*, *RABEP1* and *CLU* were associated with disease-risk regulatory regions (100).

QTL analysis is a statistical method for identifying the molecular features that are associated with a particular genotype. This can include linking non-coding genetic variants to gene expression (eQTL), enhancer activity (H3K27ac-QTL), or chromatin accessibility (caQTL) (122–124). Colocalization analysis is a statistical method that combines QTL and GWAS results to determine whether the independent associated signals at the locus are consistent with having a shared causal variant. Of note, even if a significant colocalization is found, it is still possible that the signals may be unrelated. Mendelian randomization analysis can be used to further evaluate the potential causal relationship between genetic and molecular features and the disease phenotype (97).

Studies have used gene expression analysis of human microglia to identify QTLs that colocalize with AD and PD GWAS loci (28, 107). Microglia gene expression data from large sample sizes have identified eQTLs for genes such as *BIN1*, *EPHA1-AS1*, and *PTK2B*, with an excess of colocalization with GWAS risk variants for AD, PD and inflammatory bowel disease (28, 107). Additionally, splicing QTLs have been identified for AD risk loci such as *CD33* and *MS4A4E* (28).

Recent advancements in single-cell gene expression analysis have allowed for the simultaneous examination of the genetic regulation of eight different cell types of the brain, including microglia (125, 126). By analysing data from 192 individuals across three brain regions, researchers found that microglia had the strongest genetic effect among brain cell types, likely due to their unique development (125). The strongest genetic associations for microglia were found to be related to AD, and several genes associated with the endolysosomal pathway were identified including *BIN1*, *CASS4*, *CD2AP*, *FCER1G*, *INPP5D*, *PICALM*, *RAPEB1*, *RIN3*, *TREM2*, *USP6NL* and *ZYX* (125).

Haglund et al. (126) used Mendelian randomization to investigate the relationship between cell type eQTLs and disease outcome, using single-cell RNA-seq data from 147 postmortem brain samples (126). They focused their analysis on cell-type eQTLs that were found to co-localize with GWAS risk alleles. Using this approach, the study inferred a causal link and determined the direction of the effect. They identified several genes exclusive to microglia as being putatively causal for AD, including *BIN1*, *RIN3*, *RASGEF1C*, and *JAZF1* (126). Additionally, the study found that a *PICALM* variant (rs10792832) overlapped with an open chromatin region within a microglia enhancer and was linked to the *PICALM* promoter through chromatin looping (126). Future single-cell microglia eQTL studies may identify subtype and cell state-specific eQTLs similar to eQTLs that were identified for monocytes and iPSC-derived macrophages under inflammatory conditions (127, 128).

QTL analysis has been used to study chromatin accessibility in microglia that is under genetic control (caQTL), as an indication of enhancer activity (100, 107). Kosoy et al. (100) analysed caQTL in microglia from 95 individuals and identified 5,465 caQTLs that were strongly enriched for AD and several other brain-related conditions and traits (100). Colocalization analysis with AD genetic risk loci identified *BIN1*, *EPHA1-AS1*, *PICALM*, *MS4A4E*, and *CASS4* as AD risk genes (100).

It is important to note that while colocalization or mendelian randomization of GWAS risk alleles with eQTLs and enhancer-to-gene interactions provide statistical evidence linking AD risk variants to genes, it does not definitively prove that they are causally contributing to susceptibility. While these approaches provide complementary layers of evidence for variant-to-gene-to -cell type associations, the observations could be pleiotropic or non-causal. Therefore, it is critical to validate and interpret the biological consequences of these findings through functional validation studies.

### 2.6 Post-AD GWAS: validation of risk variants and gene regulatory regions

Predictive approaches have prioritised potential causative variants that may impact gene expression. However, whether these variants have a functional impact needs to be validated. Individual risk variants can be introduced using CRISPR-mediated genome editing to generate iPSC lines with either the major or minor allele on the same genetic background to minimise effects due to genetic variability. iPSC lines with CRISPR-generated disease risk variants can be derived into microglia- like cells and tested for differences in gene expression and function, as shown for genes with coding risk variants such as *APOE* (129, 130). However, targeted genome engineering can be time-consuming and has not been extensively explored for noncoding variants. Massive Parallel Reporter Assays (MPRAs) have been used as a high-throughput approach to test the effects of multiple variants on the expression of a reporter (131). MPRAs have been leveraged to assess the effect of variants associated with neurological disorders localised to cis- and trans- regulatory elements (111, 132). Limitations of MPRAs are the loss of genomic context and the absence of variant effects on specific target genes. An additional challenge has been to perform MPRAs in microglia, which are a challenging cell type for viral-based approaches. However, initial studies have administered MPRAs to test the effect of variants at 9 AD-risk loci in K562 chronic myelogenous leukaemia lymphoblasts and SK-SY5Y human neuroblastoma cells (133) and variants at 25 AD-risk loci in HEK293 cells (106). The latter MPRA identified 29 high-confidence functional regulatory variants across 15 AD risk loci based on localisation to functional elements (106).

Validation of the gene targets for functional regulatory variants can be tested using CRISPR excision of gene regulatory regions, as demonstrated for two *BIN1* enhancers harbouring AD risk variants rs6733839 (98) and rs1302717 (106) (**Figure 2**). CRISPR interference (CRISPRi) can be used as a high-throughput approach to screen gene regulatory regions. CRISPRi directs a transcriptional repressor domain, such as the Krüppel associated box (KRAB) domain, to gene regulatory regions through fusion to a catalytically dead Cas9 (134). Cooper et al. have validated microglia gene targets for a number of MPRA functional regulatory variants using CRISPR-excision and CRISPRi, including the AD-risk genes *CR1*, *SPI1*, *CELF1*, *MS4A4E*, *RIN3*, *KNOP1*, *BIN1* and *EPHA1* (106). CRISPRi has been implemented in an inducible iPSC line that generates microglia-like cells in 8 days and has been used to screen for genes that impact cell survival, inflammation and phagocytosis and has been coupled to single-cell gene expression analysis (CROP-seq) to identify disease-associated microglia subclusters (135). While these experiments did not target noncoding gene regulatory regions, this platform shows great promise for screening further disease-associated variants in the context of microglia cellular function.

### 2.7 Post-AD GWAS: biological interpretation

Interpreting the biological consequences of GWAS risk alleles within the pathophysiology of AD is a major objective. There are several reviews that have focused on the biological interpretation of key genes in the pathogenesis of AD (69, 136). Broadly, AD risk genes are associated with lipid transport (*APOE, CLU*), transmembrane signalling (*SORL1, TREM2, CD33, MS4A6A*), and membrane and cytoskeletal dynamics (*INPP5D, PLCG2, BIN1, CASS4*).

There are several important considerations when establishing a physiologically relevant model system to study genetic risk factors. The first consideration is that genes and proteins have different functions depending on the cell (sub)type and mechanisms in which they partake. For many AD GWAS risk genes, these general functions have not been fully elucidated, or the function of these genes were studied using cultured microglia or ‘microglia-like’ cells. A major limitation of studying AD risk genes using cultured microglia is that microglia gene expression is highly dependent on the brain microenvironment and can change in culture, limiting its translational potential to *in vivo* conditions (2, 137). A second consideration is that the pathophysiological processes of AD involve a wide range of cellular and molecular disturbances including protein aggregation, reactive oxygen species, mitochondrial stress, inflammation and decreased synaptic density. It is currently challenging to model all these features simultaneously, which makes it difficult to understand the role of AD GWAS risk genes in microglia in the pathophysiology of these disorders. A third consideration is that AD is a human-specific disorder. Studies have found considerable discrepancies between AD mouse models and human AD brain tissues, suggesting that there are organism-specific differences (59, 138). Chimeric mouse models that allow for the integration of human microglia have been developed that partially circumvent these differences, but these models have shown that human and mouse microglia exhibit divergent gene expression signatures in response to amyloid beta, illustrating species-specific divergence in the response potential of microglia (139, 140).

Recent technological advances in human brain organoids that are engrafted with microglia-like cells have shown promise in addressing the limitations of previous models (141). In a recent study, Cakir et al. (142) generated human cortical organoids with microglia by using PU.1 overexpressing embryonic stem cells (142). Organoids with microglia-like cells were found to be protected against cellular damage caused by amyloid beta, when compared to organoids without microglia-like cells (142). CRISPR-mediated knockdown of AD risk genes was used to investigate the role of microglia AD GWAS genes in amyloid beta-positive organoids (142). Downregulation of *SORL1*, *BIN1* or *PICALM* altered gene expression signatures that were associated with endocytosis and affected cholesterol metabolism in amyloid beta-positive-organoids (142). Similarly, lower expression of *TREM2* and *SORL1* changed the morphology of microglia and led to an increase in cell death, suggesting that these genes play a role in the protective functions of microglia (142). However, brain organoids resemble the prenatal brain, which limits the ability of these organoids to model the mature brain (143). Therefore, developing reliable strategies to age brain organoids will be crucial for studying ageing-related disorders such as AD and PD (144, 145).

An extensive number of studies have aimed to study the role of AD GWAS risk genes often beyond the confines of microglia, using additional model systems, which have been reviewed by others (69, 146, 147). Overall, many AD-risk genes have immune-associated functions such as lipid metabolism, endolysosomal trafficking, amyloid and tau processing, and efferocytosis (34, 35, 148). Of note, many AD-risk genes also have pleiotropic functional roles in different cell types and different tissues. Hence, it is likely that AD risk genes may have functions that are not directly affected by GWAS risk alleles. Consequently, studies on the biological interpretation of these genes often result in multiple interpretations that are not always easy to unify. The above-mentioned genomics datasets and computational tools can help guide the study of disease-risk genes by more specifically determining the relevant cell subtypes and mechanisms. Nonetheless, previous studies have provided new insights into the roles of AD GWAS risk genes on disease pathophysiology. These functional studies often emphasise a critical role for differential regulation of functionally divergent splice variants and isoforms. This will be illustrated in brief for *BIN1*, *PICALM* and *CD33*.

After *APOE*, the *BIN1* locus has the largest effect sizes, and has been extensively studied. *BIN1* has 20 exons in which multiple functional splice variants (alternative mRNAs) and isoforms (alternative protein structures) might play differential roles in AD pathogenesis. *BIN1* is associated with different cell type-specific isoforms, in which the neuronal BIN1 isoform is downregulated and a ubiquitous BIN1 isoform is upregulated in AD (146, 149, 150). Functional studies have linked *BIN1* to tau neurotoxicity (146, 151), endocytic uptake of amyloid beta (146, 152, 153), and inflammation (146, 154). Many of these studies were performed in non-microglia cells, including neurons, suggesting that the *BIN1* gene is a highly pleiotropic gene with multiple important functions, some of which might not be related to the mechanisms by which the GWAS risk alleles exert their effect. Future studies should aim to disentangle the general functions of genes, from their mechanisms in the context of GWAS risk alleles. The *PICALM* locus is the third strongest AD risk locus. The *PICALM* gene has 21 exons, with multiple functional splice variants and isoforms and is expressed in multiple brain cell types. *PICALM* isoforms have been implicated in AD pathogenesis (147, 155), which may have differential roles in microglia (100), neurons (155) and endothelial cells (156). *PICALM* plays a role in processing of APP, endocytosis of amyloid beta, and propagation of tau (147, 157).

In contrast, expression of the AD GWAS risk gene *CD33* in the brain is exclusively restricted to myeloid cells including microglia (158). The AD risk locus associated with *CD33* modulates both the expression and distribution of 2 splice variants. The alternative genetic variant induces the expression of a *CD33* isoform that is absent of a ligand-binding domain (159, 160). Lower *CD33* expression is associated with decreased soluble amyloid-beta, suggesting that CD33 inhibits amyloid-beta uptake by microglia (158, 161, 162). Hence, it has been suggested that CD33 inactivation could be a putative drug target for the amelioration of AD pathogenesis.

## 3 Parkinson’s Disease

### 3.1 PD and the role of microglia in pathophysiology

PD is a progressive neurodegenerative disorder that affects movement and motor control. It is characterised by the loss of dopamine-producing neurons in a region of the midbrain called the substantia nigra pars compacta (SNc) (163, 164). The loss of dopamine in the brain leads to the development of the classic symptoms of PD, such as tremor, rigidity, bradykinesia (slowness of movement), and postural instability (165). The molecular pathological hallmarks of PD are the presence of intraneuronal protein inclusions called Lewy bodies and Lewy neurites, which are primarily composed of the protein alpha-synuclein (166, 167). While the histopathological assessment of PD requires the identification of Lewy bodies or neurites, the contribution of alpha-synuclein aggregates to the pathogenesis of PD is still not fully understood. Recent studies have highlighted the possible role of multiple other processes in the development of PD, including mitochondrial dysfunction, oxidative stress, and neuroinflammation (168, 169).

Several studies have shown that microglia exhibit ameboid morphology in the early stages of PD, indicative of functional changes, that might contribute to the inflammatory and neurodegenerative processes that occur in the brain (23). Microglia play a role in the clearance of alpha-synuclein (170, 171). Studies have also highlighted the role of microglia in the release of inflammatory cytokines mediating neuroinflammation, which is a key process in the development of PD (23). Single cell RNA-sequencing of the human midbrain revealed increased microglia numbers associated with an ameboid activation state, and increased expression of genes related to unfolded protein response and cytokine signalling (172). A dual role for microglia in PD has been suggested between pro-inflammatory and anti-inflammatory signals in the response to alpha-synuclein [reviewed in Bloem et al., 2022 (173)].

### 3.2 Genetics of PD

The genetics of PD is complex, involving both common and rare genetic factors. Familial cases of PD, where multiple family members are affected, are more likely to be caused by rare genetic variants. Studies of familial PD have identified rare mutations in several genes including *SNCA* (the gene that codes for alpha-synuclein), *LRRK2*, *PRKN*, *PARK7 (DJ-1*), and *PINK1* (174–179). GWAS for PD have identified 78 risk loci that account for 16-36% of PD heritable risk (36, 180, 181), which is in concordance with twin studies that suggest a heritability of 34-40% (182). GWASs for PD have identified common genetic variants at loci near genes that include *SNCA*, *LRRK2*, *GBA*, and *MAPT*, among others (180, 183–185). These genetic studies, both GWAS and familial, have significantly increased our understanding of the genetic landscape of PD and have provided potential therapeutic targets for the development of new treatments.

### 3.3 Post-PD GWAS: target cell types and cell states

Cell type enrichment for PD GWAS heritability has not conclusively identified a major dysregulated genetic cell type. However, studies have found enrichment of GWAS heritability for certain neuronal subtypes using mouse brain single-cell gene expression data (36). Additionally, significant enrichment of PD heritability was observed for lysosomal genes, which were found to be expressed across multiple cell types including microglia (186).

MAGMA subsequently identified PD heritability enrichment in dopaminergic, enteric neurons, and oligodendrocytes using single-cell gene expression data from the mouse nervous system (96) and human substantia nigra (187, 188). However, these findings contrast with a sLDSC regression analysis of open chromatin regions, which found PD heritability to be associated with microglia and monocytes over other brain cell types (189). Interestingly, a recent single-cell gene expression MAGMA analysis of midbrains from control and PD cases showed that microglia have the strongest enrichment of PD risk genes and that this association was increased in a disease context (172). However, genomic annotation of gene regulatory regions for many of the cell types associated the midbrain is still lacking. These findings suggest that further research is needed to fully understand the cell type-specific mechanisms underlying PD heritability.

### 3.4 Post-PD GWAS: causal variants and their mechanisms

Several studies have aimed to identify variants that are causal in GWAS risk loci and to interpret the mechanism by which these causal variants exert their effect. In one study, a 95% credible set of 190 putative causal variants for PD GWAS risk loci were identified (190). 141 of the SNPs were localised to microglia regulatory elements, which is more than for regulatory elements of other brain cell types (190). Two loci were described in more detail. At the *LRRK2* locus, two consensus SNPs were identified that overlapped with putative microglia gene regulatory regions (190). At the *FCGR2A* locus, a variant was identified that perturbs an SPIB-motif that strongly resembles the *SPI1* motif that could be bound by the PU.1 transcription factor (190).

The microglia open chromatin trained machine learning model by Corces et al, was used to identify putative causal PD SNPs (99). The model predicted that a KLF4 motif was disrupted by a variant (rs181391313) within the intron of *STAB1* at the *ITIH1* GWAS locus (99). KLF4, a transcription factor, was considered to be a likely binding partner of PU.1, suggesting that its mechanism of action may be related to decreased binding of the myeloid PU.1 transcription factor. However, whether differential binding of transcription factors by causal variants influences disease susceptibility is still a matter of speculation and requires further research.

### 3.5 Post-PD GWAS: predict risk genes and regulatory elements

Microglia PD risk genes were identified using chromatin loops generated from bulk brain tissue (H3K27ac-HiChIP) and annotated to single-cell open chromatin regions (99). For example, disease- risk variants at the *ITIH1* locus overlapped with microglia open chromatin regions and were linked by chromatin loops to the *STAB1* promoter (99). While chromatin conformation data has been generated in microglia-enriched populations (98, 100), these have not been extensively analysed in the context of PD genetic risk. However, the potential of available microglia chromatin looping data (98) was shown by linking a PD risk variant that was identified as a microglia eQTL to the *P2RY12* gene (126). Further interrogation of microglia chromatin architecture will deepen our understanding of the relationship between microglia and PD, and the genetic mechanisms that play a role in the development of the disease.

Gene expression analysis of purified human microglia has resulted in QTLs that colocalize with PD GWAS risk loci (28, 107). For example, microglia eQTLs showed colocalization with 18 PD GWAS loci, including *CHRNB*1 and *P2RY12* (28, 107). Some of these eQTLs also showed an overlap with microglia enhancers and were linked to their eQTL-genes through chromatin looping, including *P2RY12* (28, 189).

Single-nucleus eQTL analysis of eight CNS cell types identified microglia eQTLs that colocalize with PD risk loci, including *TMEM163* and *GPNMB* (125, 126, 191). In Bryois et al. (125), the familial PD gene *LRRK2* showed subthreshold colocalization in microglia (125). A subsequent single-cell gene expression study using 15 cortical samples identified a PD risk variant (rs76904798) at the *LRRK2* locus as an eQTL in microglia (191). However, CRISPR-mediated editing of the rs76904798 variant in iPSCs did not change *LRRK2* expression in iPSC-derived microglia-like cells, demonstrating the challenges of interpreting disease risk using eQTL data (191). Recently, Mendelian randomization examining the relationship between cell type eQTLs and disease outcome identified *LRRC37A*, *LRRC37A2* and *ARL17A* as putative causal genes for PD in microglia and other cell types (126).

### 3.6 Post-PD GWAS: validation and biological interpretation

In general, validation and biological interpretation of PD risk genes that are associated with microglia are in their infancy. PD risk genes are associated with immune function (*LRRK2*, *BST1*) (192, 193), lysosome signalling (*LRRK2*, *SCNA*) (194, 195), and microglia function (*P2RY12*, *GPNMB*) (196, 197). *GPNMB* has been identified in disease-associated microglia in multiple disorders (197), including AD (198, 199) and gliomas (200), which has led to speculation on the role of *GPNMB* in disease susceptibility for PD. Interestingly, a *GPNMB* knock-out in two mouse models of alpha-synuclein had no impact on the disease progression, suggesting that its mechanism is not related to alpha-synuclein uptake or metabolism (201). *P2RY12* is identified as a microglia marker gene that is highly expressed in physiological microglia (137), and downregulated in inflammatory and neurodegenerative conditions (202). *P2RY12* is a purinergic receptor that is involved in microglia motility and migration (196), and plays an important role in microglia tissue repair response (203). Other reviews have covered the biological interpretation of genetic risk genes in the pathogenesis of PD in further detail (204).

Similar to AD, novel brain organoid methods have been established and show great promise to study risk genes in PD (205, 206). These methods have established organoids with a midbrain identity that are composed of different cell types including dopaminergic neurons. For example, Smits et al. (205) used PD patient-derived iPSCs with an *LRRK2*-G2019S mutation and found disease-relevant morphological changes in dopaminergic neurons (205). In contrast, Kim et al., 206 used isogenic organoids with the same *LRRK2*-G2019S mutation and found that the thiol-oxidoreductase, *TXNIP*, is important for PD development (206). However, it should be noted that the brain organoid systems used in these studies were devoid of microglia-like cells, which could affect pathophysiological developments. The importance of microglia in PD disease progression was illustrated by George et al. (207) who found that the number of microglia and the microglia inflammatory status influences alpha-synuclein aggregation and propagation in mouse brains (207). These limited studies demonstrate that there is great potential for research into the effect of PD GWAS risk on microglia to study the pathophysiology of this severe disorder.

## 4 Discussion

In this review, we have outlined the key literature regarding microglia in AD and PD, with a focus on common genetic variants associated with these disorders. Fifteen years after the first GWAS study many genetic risk loci have been detected for the two most common neurodegenerative brain disorders, AD and PD. However, for most other neurodegenerative disorders, these studies are still relatively underpowered. Moreover, most GWASs focus on a disease versus control study design and are still in their infancy in regard to integrating disease progression, age of onset, and other phenotypic or biological markers. It is expected that more large-scale GWASs will be available in the near future, which will include deeper phenotypic information from larger cohorts. Large scale post-mortem neuropathological examinations will be crucial due to the complex relationship between manifestation of dementia and the underlying neuropathological causes.

In general, most GWASs indicate that many tissue-relevant cell types contribute in varying degrees to disease susceptibility (208). Microglia will certainly play a key role in several of these disorders, which might not be equally important across different neurodegenerative diseases. For example, GWASs for frontotemporal dementia have identified a number of loci in the vicinity of immune genes, suggesting that some of these loci might affect microglia

(209, 210). In contrast, other loci are thought to target neuronal survival and differentiation (211). Hence, context matters; determining which risk loci act on which genes and in which cell (sub)types and their impact on pathophysiology will be crucial to understand genetic risk on a locus per locus manner.

There is a clear need for improved models to recapitulate the specific context in which GWAS risk loci exert their effect. New and improved models for AD, PD and other neurodegenerative disorders are increasingly being developed, including the aforementioned brain organoid models. The number of studies that use such model systems to target GWAS risk genes and enhancers is still limited but could provide a major step forward. From the AD literature, it is becoming increasingly clear that many AD risk genes affect microglia lysosomes, phagocytosis and lipid metabolism, suggesting that the ability of microglia to process amyloid beta-aggregates might be dysfunctional. Hence, the AD GWAS findings do not undermine the amyloid hypothesis but merely expand upon it. For PD, most GWAS risk genes are not studied, and a comprehensive understanding of their mechanisms in disease susceptibility is still limited. In one study, PD risk genes were associated with lysosome activity, potentially suggesting that disrupted break-down of alpha-synuclein might be implicated.

## Author contributions

AN and IH conceived and wrote the manuscript. All authors contributed to the article and approved the submitted version.
